# Antiviral and Immunomodulatory Activity of Silver Nanoparticles in Experimental RSV Infection

**DOI:** 10.3390/v11080732

**Published:** 2019-08-08

**Authors:** Dorothea Morris, Maria Ansar, Janice Speshock, Teodora Ivanciuc, Yue Qu, Antonella Casola, Roberto P. Garofalo

**Affiliations:** 1 Division of Clinical and Experimental Immunology and Infectious Disease (CEIID), Department of Pediatrics, University of Texas Medical Branch, Galveston, TX 77555, USA; 2 Department of Biological Sciences, Tarleton State University, Stephenville, TX 76401, USA; 3 Department of Microbiology and Immunology, University of Texas Medical Branch, Galveston, TX 77555, USA

**Keywords:** respiratory syncytial virus, silver nanoparticles, AgNP, neutrophils, anti-Ly6G, antiviral, epithelial cells, G-CSF, GM-CSF, KC

## Abstract

Respiratory syncytial virus (RSV) is an important etiological agent of respiratory infection in children for which no specific treatment option is available. The RSV virion contains two surface glycoproteins (F and G) that are vital for the initial phases of infection, making them critical targets for RSV therapeutics. Recent studies have identified the broad-spectrum antiviral properties of silver nanoparticles (AgNPs) against respiratory pathogens, such as adenovirus, parainfluenza, and influenza. AgNPs achieve this by attaching to viral glycoproteins, blocking entry into the host cell. The objective of this study was to evaluate the antiviral and immunomodulatory effects of AgNPs in RSV infection. Herein we demonstrate AgNP-mediated reduction in RSV replication, both in epithelial cell lines and in experimentally infected BALB/c mice. Marked reduction in pro-inflammatory cytokines (i.e., IL-1α, IL-6, TNF-α) and pro-inflammatory chemokines (i.e., CCL2, CCL3, CCL5) was also observed. Conversely, CXCL1, G-CSF, and GM-CSF were increased in RSV-infected mice treated with AgNPs, consistent with an increase of neutrophil recruitment and activation in the lung tissue. Following experimental antibody-dependent depletion of neutrophils, the antiviral effect of AgNPs in mice treated was ablated. To our knowledge, this is the first in vivo report demonstrating antiviral activity of AgNPs during RSV infection.

## 1. Introduction

Since its discovery more than 60 years ago, respiratory syncytial virus (RSV) has been recognized as the leading cause of lower respiratory tract illness (LRTI) in infants and young children worldwide [1]. RSV, of the family *Pneumoviridae*, has a genome comprised of non-segmented negative-sense RNA that encodes eleven viral proteins [2]. Infection with RSV typically begins in the epithelium of the upper respiratory tract and rapidly descends to the lower airways by intracellular transmission [3]. It is responsible for a variety of symptoms ranging from a low-grade fever to severe bronchiolitis or pneumonia [4]. On average, there are 33.1 million new infections each year, with 3.2 million hospital admissions, and 59,600 in-hospital deaths in children less than five years of age [4,5]. Currently, Palivizumab, a monoclonal antibody, is the only prophylactic medication approved for use in infants [6,7,8]. To date, there are no effective vaccines or specific treatment options for RSV infections.

Nanomedicine is a fast-growing field that utilizes nanotechnology to enhance applications, such as pharmaceuticals, diagnostic devices, and drug delivery systems [9,10,11]. Nanomaterials range in size from 1–100 nm and are classified according to their size, shape, and biological interactions [12,13]. Silver nanoparticles (AgNPs), defined as a cluster of colloidal silver, have been utilized in experimental models of infectious diseases as engineered AgNPs display the antimicrobial properties of bulk silver, with a significant reduction in the toxic effects observed with silver ions [14,15]. The antimicrobial effects of AgNPs are accomplished by a unique physiochemical property which allows for a large surface area to volume ratio, generating more efficient contact with microorganisms and enhancing interactions with microbial proteins [16,17]. This has garnered much success on an antibacterial platform, allowing for potential alternatives to antibiotic-resistant strains of bacteria, improved wound healing, and antibacterial coatings for medical materials, such as stents, catheters and orthopedic implants [18,19]. AgNPs have also demonstrated promising antiviral capabilities with viruses, such as HIV, Tacaraibe virus, and several respiratory pathogens, including adenovirus, parainfluenza and influenza (H3N2) [20,21,22,23,24].

As an antiviral agent, AgNPs have been proposed to interfere with viral replication by two separate mechanisms. The first is by binding via sulfur-bearing residues on surface glycoproteins, preventing the attachment and entry of the virus into the host cell [23]. The second mechanism involves AgNPs crossing the cell membrane and effectively blocking cellular factors necessary for the proper assembly of viral progeny [25,26]. The RSV virion contains two surface glycoproteins (F and G) that it utilizes to gain access to the host cell, where it replicates primarily within the cytoplasm [27,28]. Therefore, AgNPs have the potential to block entry of RSV by binding of surface glycoproteins and/or inhibit the spread of RSV from within the host cell.

Based on the success of AgNPs with similar pathogens, the current study was designed to analyze the antiviral and immunomodulatory capacity of AgNPs against RSV infection, utilizing both in vitro and in vivo models. Here we show significant reductions of RSV replication in A549 and HEp-2 cell lines, as well as in experimentally infected BALB/c mice. We also demonstrate a shift in the cytokine profile within the bronchoalveolar lavage fluid (BALF) of mice inoculated with AgNP-RSV to one that supports neutrophil recruitment and activation. Upon depletion of neutrophils with anti-Ly6G, the antiviral effects of AgNPs were reversed, suggesting neutrophils as a primary mechanism to the antiviral activity presented by AgNPs in vivo. This study gives insight into the antiviral mechanisms of AgNPs and their potential therapeutic application for RSV infections.

## 2. Materials and Methods

### 2.1. RSV Preparation

RSV Long strain was grown in HEp-2 cells and purified by centrifugation on discontinuous sucrose gradients. The titer of viral pools was determined by a methylcellulose plaque assay using HEp-2 cells, as described previously [29,30,31]. Virus pools were aliquoted, quick-frozen on dry ice-alcohol, and stored at −80 °C until needed.

### 2.2. PVP-Coated Silver Nanoparticles (AgNP)

10 nm poly-vinylpyrrolidone (PVP) coated biopure silver nanospheres were purchased from NanoComposix Inc. (San Diego, CA, USA). The PVP coating was chosen for its tight association with the silver particle, making the AgNP as stable as possible in a variety of different environments. Per the manufacturer, the AgNPs used in this study have a mass concentration of 1 mg/mL with a size distribution of 8–12 nm. The AgNPs exhibited an optimal density of 155 cm^−1^ and a peak wavelength of 390 nm. Endotoxin concentrations were less than 5 EU/mL, and silver purity was 99.99%. TEM images of the AgNPs provided by the manufacturer are available upon request. For in vitro analyses, AgNPs were diluted in F12K or MEM (110 mM glutamine, 100 IU/mL penicillin, and 100 μg/mL streptomycin) to a total volume of 1 mL. For in vivo analyses, AgNPs were diluted in sterile phosphate-buffered saline (PBS) prior to mice inoculation.

### 2.3. Studies In Vitro

A549 cells, a human alveolar type II-like epithelial cell line, and HEp-2 Cells (American Type Culture Collection, Manassas, VA, USA) were grown in F12K and MEM, respectively. Media contained 10% (vol/vol) FBS, 10 mM glutamine, 100 IU/mL penicillin, and 100 μg/mL streptomycin. Confluent monolayers were infected with RSV treated with varying doses of AgNPs (0, 10, 25 or 50 µg/mL). Samples were incubated with shaking for 1 h at room temperature prior to plating. A549 cells were infected at a multiplicity of infection (MOI) of 1 for 24 h. HEp-2 cells were infected at an MOI of 0.01 for 48 h. Supernatants were aliquoted and stored at −80 °C. To evaluate viral titer, serial five-fold dilutions of infected supernatants were determined by plaque assay on HEp-2 cells under methylcellulose overlay. Plaques were visualized five days later, and viral titers were calculated as PFU/mL. Additionally, CXCL8 (IL-8) and CCL5 (RANTES) were also quantified by an enzyme-linked immunosorbent assay (ELISA) (R&D Systems, Minneapolis, MN, USA).

The toxicity of AgNPs on epithelial cells was assessed in vitro with an A549 cell line, using lactate dehydrogenase (LDH) activity as an index of cellular damage. To measure LDH activity, A549 epithelial cells were exposed to varying doses of AgNPs (0, 10, and 50 μg/mL) for 24 h. LDH in the medium was measured by colorimetric assay using a commercially available kit (Cayman Chemical, Ann arbor, MI, USA) following the manufacturer’s instructions. This assay measures cellular damage in response to chemical compounds or environmental factors using a coupled two-step reaction, as previously described [32].

### 2.4. Viral Infection of BALB/c Mice

Female, 10 to 12-week-old BALB/c mice were purchased from Jackson Laboratory (Bar Harbor, ME, USA) and housed under pathogen-free conditions in the animal research facility of the University of Texas Medical Branch (UTMB), Galveston, Tex. All care and procedures involving mice in this study were in accordance with the recommendations in the Guide for the Care and Use of Laboratory Animals of the National Institutes of Health and UTMB institutional guidelines for animal care. A mixture of Ketamine (90–150 mg/kg) and Xylazine (7.5–16 mg/kg) was administered by intraperitoneal (IP) injection for anesthesia and euthanasia. This protocol was approved by the Institutional Animal Care and Use Committee of UTMB (protocol number 9001002I).

The dosage of AgNPs was calculated based on the weight of the animals. All inoculants were incubated with shaking for 1 h at room temperature prior to inoculation. Under light anesthesia, mice were intranasally inoculated with 100 µL of sterile PBS as a mock inoculation, AgNPs (2 mg/kg or 4 mg/kg) diluted in PBS (denoted as AgNP-PBS), RSV diluted in PBS at a dose of 5 × 10^6^ PFU (denoted as RSV), or RSV mixed with AgNPs (2 mg/kg or 4 mg/kg) diluted in PBS (denoted as AgNP-RSV). Animals from all groups were evaluated on a daily basis for weight loss, illness score, and presence of respiratory symptoms. The percentage of bodyweight change was plotted over time. Clinical illness scores were visually determined by two investigators using a standardized 0–5 grading system (0-no disease, 1-slightly ruffled fur, 2-full ruffled fur, 3-ruffled fur and hunched back, 4-ruffled fur, hunched back and inactive, and 5-death). These parameters have been shown to closely correlate with lung pathology in experimental infection of mice [33,34,35].

Determination of viral copy number and *Mus musculus* MX dynamin-like GTPase 1 (Mx1) in the mouse lung was done using quantitative real-time PCR (qRT-PCR). Total RNA was extracted using a ToTALLY RNA kit (catalog number AM1910; Ambion, Austin, TX, USA). RNA samples were quantified by a NanoDrop spectrophotometer and quality was analyzed on an RNA Nano-drop by the Agilent 2100 bioanalyzer (Agilent Technologies). Synthesis of cDNA was performed with 1 µg of total RNA in a 20 µL reaction mixture by using TaqMan Reverse Transcription Reagents kit from ABI (catalog number N8080234; Applied Biosystems). Amplification was done using 1 µL of cDNA in a total volume of 25 µL using the Faststart Universal SYBR green Master Mix (Roche Applied Science #04913850001). The RSV N-specific reverse transcriptase (RT) primer contained a tag sequence from the bacterial chloramphenicol resistance (Cm^r^) gene to generate the cDNA, because of self-priming exhibited by RSV RNA. To detect the RSV genome (-) strand, we used RSV N RT primer 5′-CTGCGATGAGTGGCAGGCACTACAGTGTATTAGACTTRACAGCAGAAG-3′. For PCR assays, we used RSV tag primer CTGCGATGAGTGGCAGGC and primer RSV P GCATCTTCTCCATGRAATTCAGG. To detect Mx1, the mRNA sequence reported under GenBank accession number NM_010846 was used to design amplification primers for qRT-PCR assays. 18S RNA was used as a housekeeping gene for normalization. PCR assays were run in the ABI Prism 7500 Sequence Detection System. Triplicate cycle threshold (*C_T_*) values were analyzed in Microsoft Excel by the comparative *C_T_* (ΔΔ*C_T_*) method according to the manufacturer’s instructions (Applied Biosystems). The amount of target (2^−^^ΔΔCT^) was obtained by normalization to the endogenous reference (18S) sample. RNA isolation, primer design, and qRT-PCR assays were performed at the Molecular Genomics Core, UTMB, Galveston [36,37].

### 2.5. Airway Obstruction

Airway obstruction was measured in unrestrained mice at days one and five post-infection (p.i.) using whole-body barometric plethysmography (Buxco Electronics, Troy, NY, USA) to record enhanced pause (Penh), as previously described [33]. Penh is a dimensionless value that represents a function of the ratio of peak expiratory flow to peak inspiratory flow and a function of the timing of expiration. To establish baseline airway obstruction values, mice were acclimatized to the chambers for five minutes, and respiratory activity was recorded for five minutes. This protocol was designed by Buxco Electronics, and the laboratory staff was trained by the company on the use of this protocol.

### 2.6. Bronchoalveolar Lavage (BAL)

At days one or five p.i., mice were euthanized with an IP injection of ketamine and xylazine followed by exsanguination via the femoral vessels. An incision was made in the trachea, through which the lungs were flushed twice with 1 mL of cold sterile PBS to obtain BAL fluid (BALF). The chest cavity then was opened for lung collection. Total cell counts were determined by trypan blue staining, followed by counting of viable cells using a hemocytometer. Additionally, 100 µL of BALF was spun onto glass cytocentrifuge slides and stained with H&E (Hema 3 stain, Fisher Scientific) for differential cell counts. The remaining BALF was centrifuged and supernatants were collected, and stored at −80 °C until needed for further assays.

### 2.7. Measurement of Cytokines, Chemokines, Interferon, Total Protein and Elastase

Cytokines, chemokines, interferons, and elastase were all measured using BALF collected at day one p.i. Total proteins were measured using BALF collected at days one and five p.i. Levels of cytokines and chemokines in the BALF were determined with a Bio-Plex Pro Mouse Group I 23-plex panel (BioRad Laboratories, Hercules, CA, USA). Interferon (IFN)-α and IFN-β were measured by ELISA, following the manufacturer’s protocol (PBL Biomedical Laboratories, Piscataway, NJ, USA). Total protein concentrations were determined using the Bradford method (BioRad Laboratories, Hercules, CA, USA). Neutrophil elastase was measured using a neutrophil elastase ELISA kit (R&D Systems, Minneapolis, MN, USA). Absorbance for all microplate assays was measured on a SpectraMax 190 microplate reader (MDS Analytical Technologies, Sunnydale, CA, USA).

### 2.8. Neutrophil Depletion in BALB/c Mice

In a separate set of experiments, 10 to 12-week-old female BALB/c mice were depleted of neutrophils with an IP injection of 200 μg anti-Ly6G (Clone 1A8; Bio X Cell, West Lebanon, NH, USA) in a final volume of 100 μL, 12 h prior to infection. Anti-Ly6G was diluted in PBS immediately before being administered to mice. Control mice received the same volume of PBS via IP injection 12 h prior to infection. For infection, mice were intranasal inoculated with 100 µL RSV diluted in PBS at a dose of 5 × 10^6^ PFU or RSV treated with AgNPs (2 mg/kg or 4 mg/kg) diluted in PBS. Lungs were collected at day five p.i. for determination of viral titer CPE plaque assay using lung homogenate (see Section 2.3) and by qRT-PCR.

### 2.9. Statistical Analysis

The data were analyzed by a one-way ANOVA followed by Tukey’s post hoc test for samples with unequal variances (GraphPad Prism 7; GraphPad Software, Inc., San Diego, CA, USA). Results are expressed as mean ± SEM for each experimental group unless stated otherwise, and *p* ≤ 0.05 value was selected to indicate significance.

## 3. Results

### 3.1. AgNPs Reduce RSV Replication in Epithelial Cell Lines

**Figure 1:**
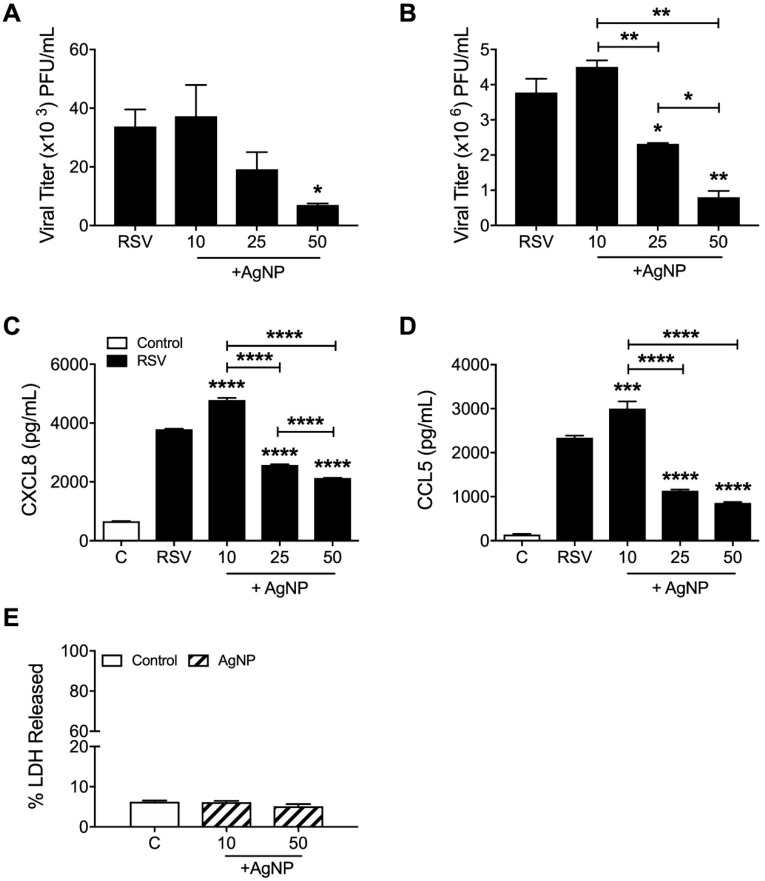
Silver nanoparticles (AgNPs) decreased respiratory syncytial virus (RSV) replication in epithelial cell lines. (**A**) A549 cells were infected with RSV or AgNP-RSV (0, 10, 25, or 50 μg/mL) at a multiplicity of infection (MOI) of 1 and (**B**) HEp-2 cells were infected with RSV or AgNP-RSV at an MOI of 0.01. Supernatants of infected cells were collected as described in the methods. Viral replication was determined by plaque assay and (**C**) CXCL8 and (**D**) CCL5 levels were determined by enzyme-linked immunosorbent assay (ELISA). (**E**) The toxicity of AgNPs (0, 10, and 50 μg/mL) on A549 cells was determined using LDH as an index of cellular damage. Data are expressed as mean ± SEM and is representative of three independent experiments. Significant results as compared to the RSV control are marked with asterisks, and additional comparisons between groups are indicated with brackets (* for *p* ≤ 0.05, ** for *p* ≤ 0.01, *** for *p* ≤ 0.001, **** for *p* ≤ 0.0001).

The effect of AgNPs on RSV infection was assessed in vitro by plaque assay in two epithelial cell lines, A549 (MOI: 1; Figure 1A) and HEp-2 (MOI: 0.01; Figure 1B). Following incubation of RSV with AgNPs (0, 10, 25, and 50 µg/mL), both cell lines demonstrated significant dose-dependent reductions in RSV replication. The dose of 50 µg/mL AgNP was the most effective in either cell type with a decrease of RSV replication by 79% in A549 cells and 78% in HEp-2 cells. This decrease was associated with a significant reduction in CXCL8 (Figure 1C) and CCL5 (Figure 1D) secretion by RSV-infected cells. Interestingly, the lowest dose of AgNP (10 µg/mL) induced a small increase in viral replication, coupled with increase secretion of CXCL8 and CCL5 (Figure 1A–D).

In addition to viral replication, the potential toxicity of AgNPs on A549 cells was also assessed using LDH as an index of cellular damage. There were no notable cytotoxic effects following 24 h of exposure to either the lowest or highest dose of AgNPs (10 or 50 μg/mL) (Figure 1E).

### 3.2. AgNPs Reduce RSV Replication in the Lung Tissue of Experimentally Infected Mice 

**Figure 2:**
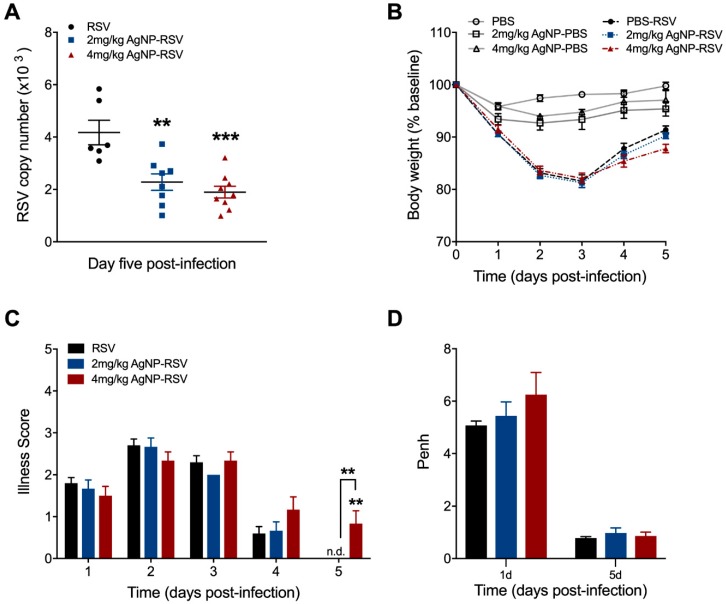

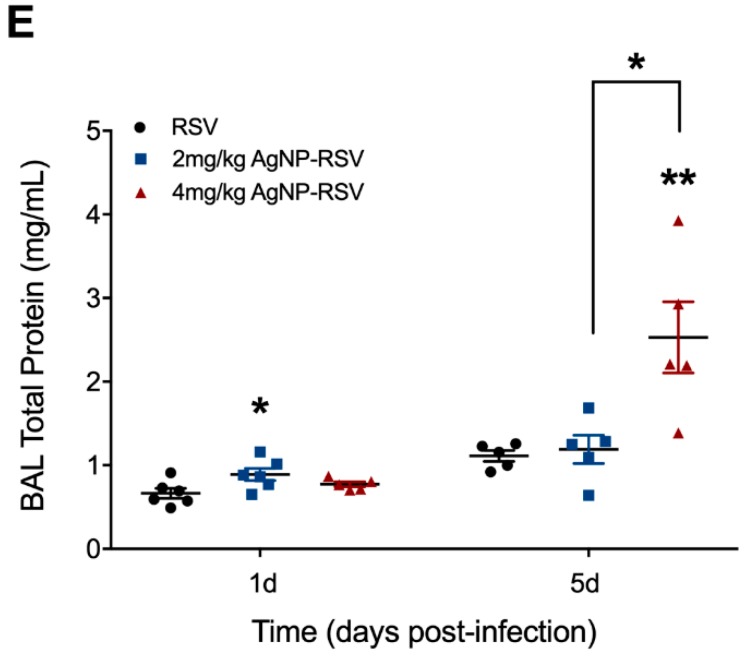
AgNPs decreased RSV replication in the lung tissue of experimentally infected mice. Under light anesthesia, mice were intranasally inoculated with either phosphate-buffered saline (PBS), AgNP-PBS (2 mg/kg or 4 mg/kg), RSV (5 × 10^6^ PFU) or AgNP-RSV. Lung tissue collected at day five p.i. was measured for (**A**) viral titer by qRT-PCR. Over the five-day infection period, mice were monitored daily for changes in (**B**) bodyweight and (**C**) illness score. (**D**) Airway obstruction, represented by baseline Penh, was assessed by unrestrained plethysmography (Buxco Electronics, Inc., Sharon, CT, USA). (**E**) Total protein concentrations of the bronchoalveolar lavage fluid (BALF) at days one and five p.i. were determined using the Bradford method (BioRad Laboratories, Hercules, CA, USA). Data are expressed as mean ± SEM (n ≥ 5 mice) and is representative of three independent experiments. Significant results as compared to the respective control are marked with asterisks, and additional comparisons between groups are indicated with brackets (* for *p* ≤ 0.05, ** for *p* ≤ 0.01, *** for *p* ≤ 0.001, **** for *p* ≤ 0.0001). Not detected (n.d).

To understand the role of AgNPs in the context of RSV infection in vivo, BALB/c mice were intranasally inoculated with PBS, AgNP-PBS (2 mg/kg or 4 mg/kg), RSV (5 × 10^6^ PFU), or AgNP-RSV. Lung tissue was collected at day five p.i. to evaluate RSV copy number by qRT-PCR. Mice treated with AgNP-RSV had significant reductions in RSV copy number as compared to the RSV untreated mice (Figure 2A). Of the two AgNP doses, 4 mg/kg AgNP was most significant, with a decrease of 55%. The dose of 2 mg/kg AgNP elicited a reduction of 45%.

Over the course of the disease, mice were monitored daily for changes in clinical parameters (i.e., bodyweight loss and illness score). Mice inoculated with AgNP-PBS did not display any signs of disease or weight loss over the five-day monitoring period, indicating that AgNPs per se do not lead to clinical illness in mice. Mice inoculated with either dose of AgNP-RSV did not differ in bodyweight loss as compared to RSV untreated mice (Figure 2B). Mice inoculated with 4 mg/kg AgNP-RSV demonstrated a minor increase in illness score only at day five p.i. (Figure 2C). No other group had any significant changes in illness score over the five-day period.

Next, to assess the effects of AgNPs on pulmonary function, airway obstruction was analyzed by whole-body plethysmography (Buxco Electronics, Inc., Sharon, CT, USA) and expressed as enhanced pause (Penh). Mice inoculated with either dose of AgNP-PBS had no notable changes to baseline Penh at day one or day five p.i. (data not shown). Mice inoculated with AgNP-RSV showed an increasing trend in baseline Penh values, but were statistically similar to the RSV untreated mice at both time points (Figure 2D). We also evaluated the concentration of total protein as a marker of epithelial damage and increased vascular permeability. At day 1 p.i., mice inoculated with 2 mg/kg AgNP-RSV demonstrated an average value of 0.89 mg/mL, which was a marginal increase compared to the RSV untreated group that demonstrated total protein levels of 0.66 mg/mL (Figure 2E). Mice inoculated with 4 mg/kg AgNP-RSV had total protein levels comparable to the RSV untreated mice. At day 5 p.i., mice inoculated with 4 mg/kg AgNP-RSV demonstrated a significant increase in total protein concentration with a value of 2.53 mg/mL as compared to 1.11 mg/mL for RSV untreated mice. Mice inoculated with 2 mg/kg AgNP-RSV had total protein levels comparable to the RSV untreated mice (Figure 2E).

### 3.3. AgNPs Decrease Many RSV-Induced Cytokines and Chemokines, While Increasing Those Associated with Neutrophil Recruitment and Activation

**Figure 3:**
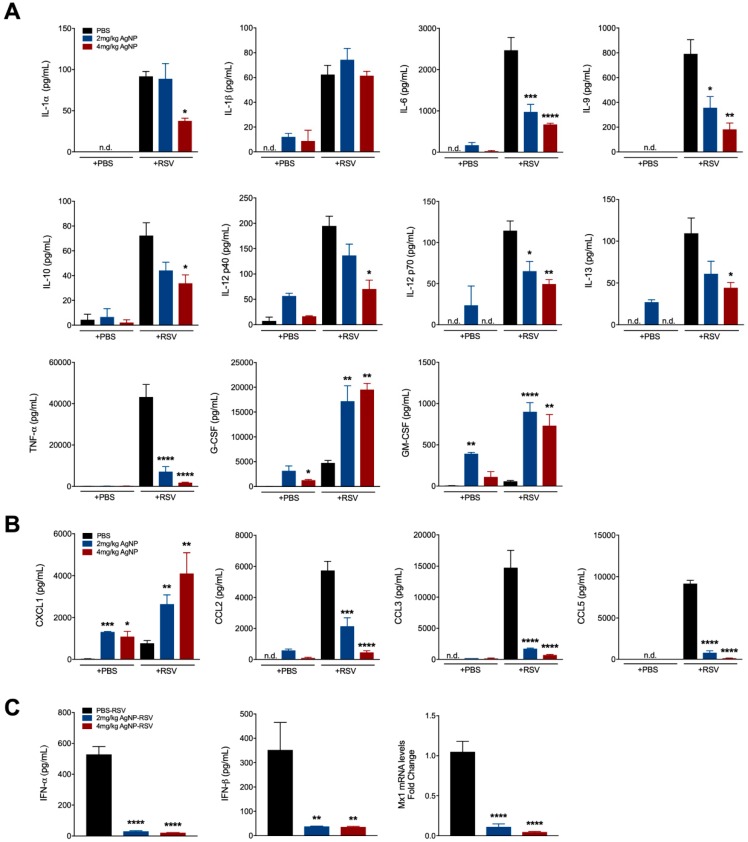
AgNPs decrease viral-induced cytokines, while increasing those associated with neutrophil recruitment and activation. BALF was collected at day one p.i. and analyzed for (**A**) cytokines, (**B**) chemokines and (**C**) type-I IFNs by Bio-plex, ELISA, and qRT-PCR analysis. Data are expressed as mean ± SEM (n ≥ 6 mice) and is representative of two independent experiments. Significant results as compared to the respective control are marked with asterisks (* for *p* ≤ 0.05, ** for *p* ≤ 0.01, *** for *p* ≤ 0.001, **** for *p* ≤ 0.0001). Not detected (n.d).

To investigate the immunomodulatory effects of AgNPs during RSV infection, cytokine and chemokine concentrations of the BALF at day one p.i. were evaluated by a multiplex cytokine array. In mice inoculated with AgNPs, regardless of infection status, G-CSF and GM-CSF were significantly increased (Figure 3A). Conversely, inflammatory and immunomodulatory cytokines, such as interleukin (IL)-1*α*, IL-6, IL-9, IL-10, IL-12p40, IL-12-p70, IL-13, and TNF-*α* were significantly decreased in mice inoculated with either dose of AgNP-RSV (Figure 3A). In addition to G-CSF and GM-CSF, the chemokine CXCL1 (KC) was significantly increased in all mice inoculated with AgNPs, regardless of infection status. Chemokines associated with viral replication, such as CCL3 (MIP-1*α*) and CCL5 (RANTES), were significantly decreased (Figure 3B).

To assess type-I IFN production, an ELISA (PBL Biomedical Laboratories, Piscataway, NJ, USA) was conducted at day one p.i. using the BALF of mice inoculated with AgNP-RSV and compared with RSV untreated mice. This was further supported by testing for the Mx1 gene within the lung tissue by qRT-PCR. IFN-*α*, IFN-*β*, and Mx1 were all significantly decreased following inoculation with either dose of AgNP-RSV as compared to the RSV untreated mice (Figure 3C).

### 3.4. AgNPs Increase and Maintain Neutrophil Cell Counts in the BALF, Regardless of Infection Status

**Figure 4:**
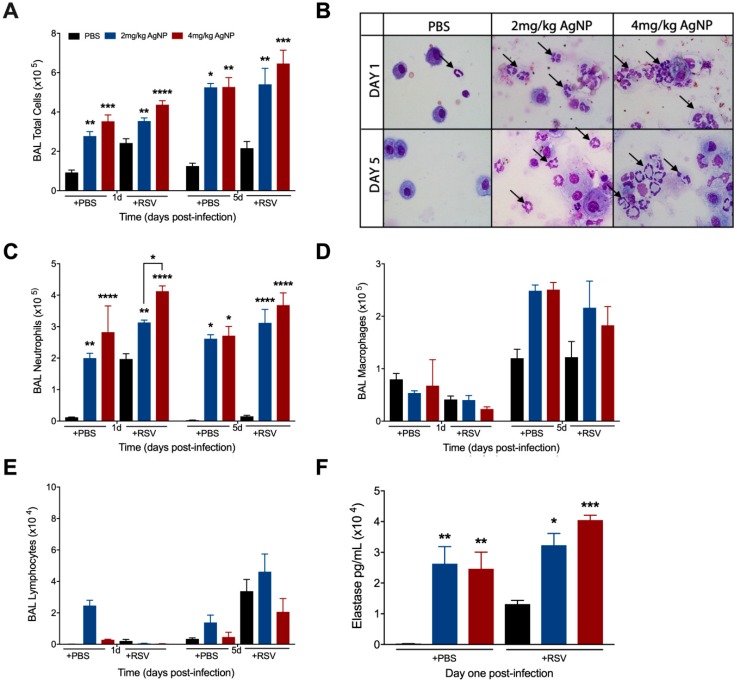
AgNPs increase recruitment and activation of neutrophils to the lung, regardless of infection status. Mice were inoculated with PBS, AgNP-PBS (2 mg/kg or 4 mg/kg), RSV (5 × 10^6^ PFU) or AgNP-RSV. At days one and five p.i., BALF was collected from all groups and used to obtain (**A**) total cell counts, as well as differential cell counts consisting of (**C**) neutrophils, (**D**) macrophages, and (**E**) lymphocytes. (**B**) BALF samples collected at days one and five p.i. from PBS untreated and AgNP-PBS mice were spun on glass slides, stained with H&E, and observed under a light microscope at 40× magnification. The black arrows indicate neutrophils. (**F**) Neutrophil elastase concentrations were measured by ELISA using BALF collected at day one p.i. Data are expressed as mean ± SEM (n ≥ 6 mice) and is representative of three independent experiments. Significant results as compared to the respective control are marked with asterisks, and additional comparisons between groups are indicated with brackets (* for *p* ≤ 0.05, ** for *p* ≤ 0.01, *** for *p* ≤ 0.001, **** for *p* ≤ 0.0001).

To determine whether AgNP treatment affected the cellular composition, BAL samples were collected from inoculated mice at days one and five p.i. for total and differential cell counts. The total cell count was significantly greater in the BALF of all AgNP inoculated mice at days one and five, regardless of infection status (Figure 4A). Differential cell counts revealed this increase to be primarily due to a significant increase in the number of neutrophils at both time points (Figure 4B,C). Macrophage cell counts were unaffected in either group at day one p.i. and had an increasing trend in cell count at day five p.i., regardless of infection status (Figure 4D). Lymphocyte cell counts were comparable to the respective controls at both days one and five p.i. (Figure 4E).

Next, we wanted to understand if the neutrophils present in the BALF of mice inoculated with either dose of AgNPs were activated using elastase as a marker of neutrophil activation. BALF collected at day one p.i. demonstrated significant increases in elastase in all AgNP inoculated mice, regardless of infection status. A dose-dependent increase in elastase production was observed in AgNP-RSV treated mice as compared to RSV untreated mice (Figure 4F).

### 3.5. Neutrophils Are a Primary Mechanism of the Antiviral Activity by AgNPs in RSV-Experimentally Infected Mice

**Figure 5:**
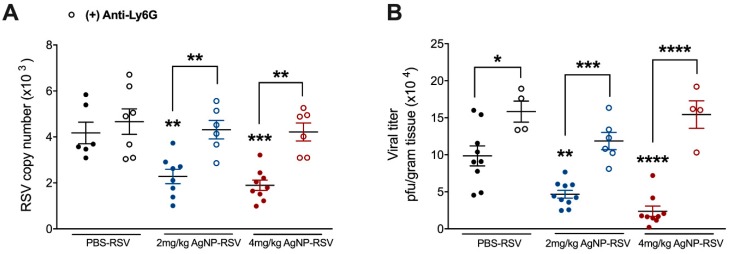
Neutrophil depletion results in a reversal of the antiviral effect noted in neutrophil immunocompetent AgNP-RSV mice. Following injection with anti-Ly6G clone 1A8, lung tissue was collected at day five p.i. for evaluation of (**A**) viral copy number by qRT-PCR and for (**B**) viral titer using lung homogenate in a viral plaque assay. Data are expressed as mean ± SEM (n ≥ 4 mice) and is representative of two independent experiments. Significant results as compared to the RSV control are marked with asterisks, and additional comparisons between groups are indicated with brackets (* for *p* ≤ 0.05, ** for *p* ≤ 0.01, *** for *p* ≤ 0.001, **** for *p* ≤ 0.0001).

Some studies have shown AgNPs to increase neutrophil cell counts within the BALF of treated mice, but few have investigated their activity following recruitment [38,39]. Neutrophils have also been suggested to have antiviral capabilities during RSV infection, leading us to evaluate the function of neutrophils in AgNP-RSV treated mice. Mice were depleted of neutrophils with an injection of anti-Ly6G clone 1A8 12 h prior to inoculation with RSV (5 × 10^6^ PFU), or AgNP-RSV. In neutrophil-depleted mice inoculated with 2 mg/kg AgNP-RSV or 4 mg/kg AgNP-RSV, the viral copy number at day five p.i. was found to be similar to the viral copy number in neutrophil-depleted RSV untreated mice, indicating a reversal of the antiviral effect noted in immunocompetent mice (Figure 5A). This was further supported by a viral plaque assay using the lung homogenate of infected mice (Figure 5B). These results support neutrophils as a primary mechanism of the antiviral activity generated by AgNPs against experimental RSV infection in mice.

## 4. Discussion

RSV accounts for nearly 7% of deaths among infants and young children worldwide, second only to malaria [40]. Despite this, there is still no effective therapeutic or licensed vaccine currently available for the treatment of RSV [6]. This has created a high demand for new antivirals and/or therapeutic agents for the treatment and control of RSV infections. The aim of this study was to evaluate the antiviral efficacy and immunomodulatory capacity of AgNPs during RSV infection, both in vitro and in vivo.

In our cell culture analysis, we found a significant dose-dependent reduction of RSV replication in both HEp-2 and A549 cell lines, with the strongest antiviral effect elicited by a dose of 50 μg/mL AgNP. In addition, cells exposed to this dose of AgNPs were found to release levels of LDH similar to that of the control, demonstrating that AgNPs at the dose of 50 μg/mL are not toxic to epithelial cells. The effectiveness of 50 μg/mL AgNPs in epithelial cell lines is in agreement with work previously reported examining the effects of AgNPs on influenza strains H1N1 and H3N2 [24,41]. To confirm our findings in vivo, BALB/c mice were inoculated with AgNP-RSV, and lung tissue was collected at day five p.i. to evaluate viral titer. Inoculation with AgNP-RSV resulted in significant reductions in viral titer as compared to RSV untreated mice. This demonstrates, for the first time, the effectiveness of AgNPs against experimental RSV infection in mice.

The mechanism to the antiviral effect against RSV in epithelial cell lines may be due to be the attachment of AgNPs to surface glycoproteins [22,23,42]. By doing so, AgNPs would interfere with RSV’s ability to initiate attachment with the proper receptors, preventing fusion of the virus to the host cell. This would leave RSV in the extracellular space where it is unable to propagate, resulting in the reduction of syncytia formation seen in our plaque assays [28,43,44]. The basis of this mechanism has been investigated with other RNA viruses, such as human immunodeficiency virus type-1 (HIV-1). AgNPs were shown to directly associate with the surface glycoprotein gp120, preventing HIV-1 from interacting with host receptors [45]. The gp120 glycoprotein has also been found to have some structural similarities with the RSV-F surface glycoprotein, supporting the hypothesis of a direct association of AgNPs with RSV [42,46,47]. Interestingly, exposure of 10 μg/mL AgNP to RSV in A549 and HEp-2 cell lines resulted in a slight increase in RSV replication and secretion of CXCL8 and CCL5. Though it is not clear at the present time why a low dose of AgNPs would have such an impact on viral parameters, we believe that insufficient coating of the virus with AgNPs would allow the virus to continue infecting epithelial cells. This enhancement of replication was not appreciated with increasing doses of AgNPs, suggesting that RSV virions are more efficiently coated with higher doses of AgNPs (Figure 1A,B).

Although a potential direct antiviral mechanism has been previously described, cell culture lacks the interaction of a complex immune system. Therefore, to elucidate potential antiviral mechanisms due to AgNPs in vivo, we investigated the cytokine, and cellular composition of the BALF collected from inoculated mice. AgNP-RSV infected mice were found to have significant reductions in many pro-inflammatory and viral associated markers, such as IL-6, TNF-α, CCL5 (RANTES), and type I IFNs. Additionally, significant reductions were also noted with IL-1α, IL-9, IL-10, IL-12p40, IL-12p70, IL-13, CCL2 (MCP-1), and CCL3 (MIP-1α). Previous data in the field of RSV has suggested that a downregulation in pro-inflammatory cytokines, such as TNF-α corresponds with significantly improved clinical disease (i.e., bodyweight loss, illness score, airway obstruction) [48,49,50,51]. Conversely, our study demonstrated no significant changes to clinical parameters in AgNP-RSV inoculated mice, despite significant decreases in an array of RSV-induced cytokines. Upregulations in cytokines and chemokines responsible for neutrophil recruitment and/or activation (i.e., elastase, CXCL1, G-CSF, and GM-CSF) have been linked to heightened airway inflammation and RSV bronchiolitis [52,53]. As shown in Figure 3, CXCL1, G-CSF, and GM-CSF were all found to be significantly increased following AgNP inoculation. Therefore, the beneficial effects typically associated with reductions in pro-inflammatory cytokines are likely counteracted by the strong presence of these neutrophil associated cytokines.

Consistent with increases in cytokines associated with neutrophil recruitment, the number of neutrophils in the BALF was also found to be significantly increased. These findings are in agreement with other reports demonstrating an upregulation of neutrophils in many rodent models following inoculation with AgNPs [38,39]. The mechanism to this neutrophilia is believed to be an initial stimulation of macrophages to release CXCL1 (KC), but the role of neutrophils during AgNP exposure is still poorly understood [54]. RSV is also known to induce neutrophilia during the early stages of the disease, but the activity of neutrophils during RSV infection has only recently begun to be properly elucidated, and reports remain contradictory [55,56,57,58,59,60,61]. Therefore, to evaluate the role of neutrophils following AgNP-RSV exposure, we depleted mice of neutrophils using an injection of anti-Ly6G. AgNP-RSV treated mice that had been depleted of neutrophils were found to have viral titers similar to RSV untreated neutrophil-depleted mice, demonstrating a reversal of the antiviral effect observed in AgNP-RSV neutrophil immunocompetent mice. These findings suggest AgNPs modulate the function of neutrophils in RSV infection to enhance their antiviral activity.

Toxicity studies of AgNPs in the lung have been performed in a variety of rodent models and have shown overall that AgNPs induce minor airway mucosa thickening and cellular infiltration (primarily neutrophils), but generate no major alterations to lung function, even following 28 days of continuous exposure [16,62,63,64,65]. This is consistent with our findings, which demonstrate a sizable neutrophil influx to the BAL fluid through day five in mice inoculated with PBS-AgNPs, but without evidence of clinical disease (i.e., bodyweight-loss, illness score, airway obstruction). Similarly, although both doses of AgNPs resulted in significantly increased neutrophil recruitment/activation in RSV-infected mice, clinical parameters were comparable to those observed in RSV-infected untreated mice at the peak of disease (day 2–4). At the time of recovery (day 5), we observed some delay in the regaining of bodyweight and clinical disease along with higher total protein content in the BAL only in the group of infected mice that had received the higher dose of AgNPS (4 mg/kg), a result of the higher number of activated neutrophils in the BALF (Figure 2) as previously suggested [56]. However, mice that received the 4 mg/kg AgNP dose also exhibited the largest reduction in RSV lung titers, as well as in all pro-inflammatory cytokines (i.e., TNF-α, IL-6), suggesting that AgNPs potentiate the neutrophil anti-RSV activity as their major antiviral function in the experimental mouse model.

In conclusion, our study demonstrates that AgNPs effectively reduce RSV replication and production of pro-inflammatory cytokines in epithelial cell lines and in mouse lung. In the mouse model, the antiviral activity appears to be mediated to a large extent by neutrophils, which are recruited in higher number to the airways and activated via a neutrophil-specific program of cytokines that include CXCL1, G-CSF, and GM-CSF. These findings contribute to the understanding of AgNP bioactivity in the lung while providing insights on the role that neutrophils play in the host response against infections caused by RSV.
